# Digital variance angiography allows about 70% decrease of DSA-related radiation exposure in lower limb X-ray angiography

**DOI:** 10.1038/s41598-021-01208-3

**Published:** 2021-11-08

**Authors:** Marcell Gyánó, Márton Berczeli, Csaba Csobay-Novák, Dávid Szöllősi, Viktor I. Óriás, István Góg, János P. Kiss, Dániel S. Veres, Krisztián Szigeti, Szabolcs Osváth, Ákos Pataki, Viktória Juhász, Zoltán Oláh, Péter Sótonyi, Balázs Nemes

**Affiliations:** 1 The Heart and Vascular Center, Semmelweis University, Városmajor utca 68, Budapest, 1122 Hungary; 2 Kinepict Health Ltd, Budapest, Hungary; 3 Department of Biophysics and Radiation Biology, Semmelweis University, Budapest, Hungary; 4 Department of Vascular Surgery, Hungarian Defence Forces Medical Centre, Budapest, Hungary

**Keywords:** Radiography, Peripheral vascular disease

## Abstract

Our aim was to investigate whether the previously observed higher contrast-to-noise ratio (CNR) and better image quality of Digital Variance Angiography (DVA) - compared to Digital Subtraction Angiography (DSA) - can be used to reduce radiation exposure in lower limb X-ray angiography. This prospective study enrolled 30 peripheral artery disease patients (mean ± SD age 70 ± 8 years) undergoing diagnostic angiography. In all patients, both normal (1.2 µGy/frame; 100%) and low-dose (0.36 µGy/frame; 30%) protocols were used for the acquisition of images in three anatomical regions (abdominal, femoral, crural). The CNR of DSA and DVA images were calculated, and the visual quality was evaluated by seven specialists using a 5-grade Likert scale. For investigating non-inferiority, the difference of low-dose DVA and normal dose DSA scores (DVA30-DSA100) was analyzed. DVA produced two- to three-fold CNR and significantly higher visual score than DSA. DVA30 proved to be superior to DSA100 in the crural region (difference 0.25 ± 0.07, *p* < 0.001), and there was no significant difference in the femoral (− 0.08 ± 0.06, *p* = 0.435) and abdominal (− 0.10 ± 0.09, *p* = 0.350) regions. Our data show that DVA allows about 70% reduction of DSA-related radiation exposure in lower limb X-ray angiography, providing a potential new radiation protection tool for the patients and the medical staff.

## Introduction

Radiation protection efforts are critically important in the current radiological procedures. Conventional fluoroscopy and X-ray angiography along with diagnostic CT imaging cumulatively expose patients to an increased risk of stochastic and deterministic radiation injuries and long-term radiation-induced complications^1,2^. Due to everyday exposure, catheter lab and hybrid operating room staff are also exposed to both stochastic and deterministic injuries, which might lead to the development of oncological disorders^3^ and cataract^4^, respectively. The demand for minimally invasive procedures is gradually increasing as the procedures are evolving and evidence-based international guidelines are broadening the indication field for the vascular, oncological and other interventional procedures, such as prostatic artery embolization^5^ or transarterial chemoembolization^6^. Therefore, the demand for radiation protection measures^7,8^ is increasing in the medical device research and development area^9^. Apart from appropriate shielding instruments and limited working hours for staff^10^, the aspects of the As Low As Reasonably Achievable (ALARA) principle are also associated with radiation dose management measures during image acquisition^11–13^, and critical for both the patients and the medical staff.

Digital Subtraction Angiography (DSA) is the reference-standard image processing method for vessel visualization during interventional procedures. A recently published study using an anthropomorphic phantom investigated the radiation load of the medical staff, and found that DSA results in about a 30-fold increase in radiation dose to the operating physician compared to low dose fluoroscopy^14^. As angiogram and roadmap acquisitions are essential parts of all interventional procedures, research is also focused on decreasing the amount of radiation needed to obtain these images. A study published by de Ruiter et al. compared the mean air kerma (AK) dose rates of DSA and fluoroscopy protocols. The results revealed that the fluoroscopic protocol has a predicted AK of 0.39 mGy/s (or 85.5 min until the 2-Gy skin threshold is reached). While using comparable settings but switching the acquisition to a DSA with a "2 frames per second" protocol, the predicted AK was 6.6 mGy/s (or 5.0 min until the 2-Gy threshold is reached), showing a 17-fold higher AK for DSA. Based on the results, the authors recommended to reduce the number DSA runs, the amount of time and total fps necessary to a minimum^15^. These data indicate that the reduction of DSA-related radiation load might provide a very robust radiation protection measure.

Digital Variance Angiography (DVA) is a recently developed medical image processing method. The technology is based on the principles of kinetic imaging^16^, and calculates the standard deviation for each pixel position in an image series. Consequently, the contrast media-induced changes are amplified but the background noise is suppressed, and the image quality is greatly improved. In recent clinical studies DVA provided higher contrast-to-noise ratio (CNR) and better subjective image quality than conventional DSA, both with iodinated contrast media (ICM)^17,18^ and carbon-dioxide^19^. This significant quality reserve might provide opportunity for contrast media and/or radiation dose reduction, further decreasing the risk of interventional procedures. The aim of this proof-of-concept study was to investigate whether the previously observed quality reserve of DVA can be converted to substantial radiation dose reduction in lower limb X-ray angiography.

## Materials and methods

The study was approved by the National Institute of Pharmacy and Nutrition (reference number OGYÉI/2830/2017). All study activities were in accordance with the ethical standards of the national Medical Research Council and with the Helsinki Declaration. Written informed consent was obtained from all participants included in the study.

### Patient selection

This prospective study enrolled 30 Peripheral Artery Disease (PAD) patients (mean age 70 years, range 52–85 years; 10 females, mean age 73 years, range 55–85 year; and 20 males, mean age 69 years, range 52–85) undergoing diagnostic lower limb X-ray angiography between April and July 2019 at the Heart and Vascular Center (Semmelweis University, Budapest, Hungary). For detailed demographics see Table 1. Participants were selected in a consecutive manner based on the eligibility criteria. Inclusion criteria were elective patients with symptomatic PAD (Fontaine IIb-IV), glomerular filtration rate over 60 ml/min/1.73m^2^, age over 50 years. Exclusion criteria were acute myocardial infarction, atrioventricular block, severe heart, liver or renal failure and patients with acute lower extremity arterial occlusion. The number of patients was determined on the basis of a Food and Drug Administration Guideline developed for the concurrence testing of X-ray imaging devices^20^. Patients received clinical standard of care and all procedures were performed according to the institutional protocol.

**Table 1:** Patient demographics.

Parameter	Age (y)	Height (cm)	Weight (kg)	BMI (kg/m^2^)	eGFR (ml/ min/1.73m^2^)	ICM use (ml)
**All patients (n = 30)**						
Mean ± SD	70 ± 8	170 ± 10	80 ± 14	27.9 ± 4.8	80 ± 11	102 ± 10
Median	71	170	82	28.3	86	102
**Female patients (n = 10)**						
Mean ± SD	73 ± 9	160 ± 6	76 ± 14	29.9 ± 6.3	74 ± 12	96 ± 5
Median	74	161	81	30.3	70	98
**Male patients (n = 20)**						
Mean ± SD	69 ± 8	175 ± 7	82 ± 14	26.9 ± 3.6	84 ± 9	106 ± 10
Median	70	177	84	27.4	88	105

The ICM column shows the total procedural ICM use. *BMI* body mass index, *ICM* iodinated contrast media, *SD* standard deviation.

### Study design

**Figure 1:**
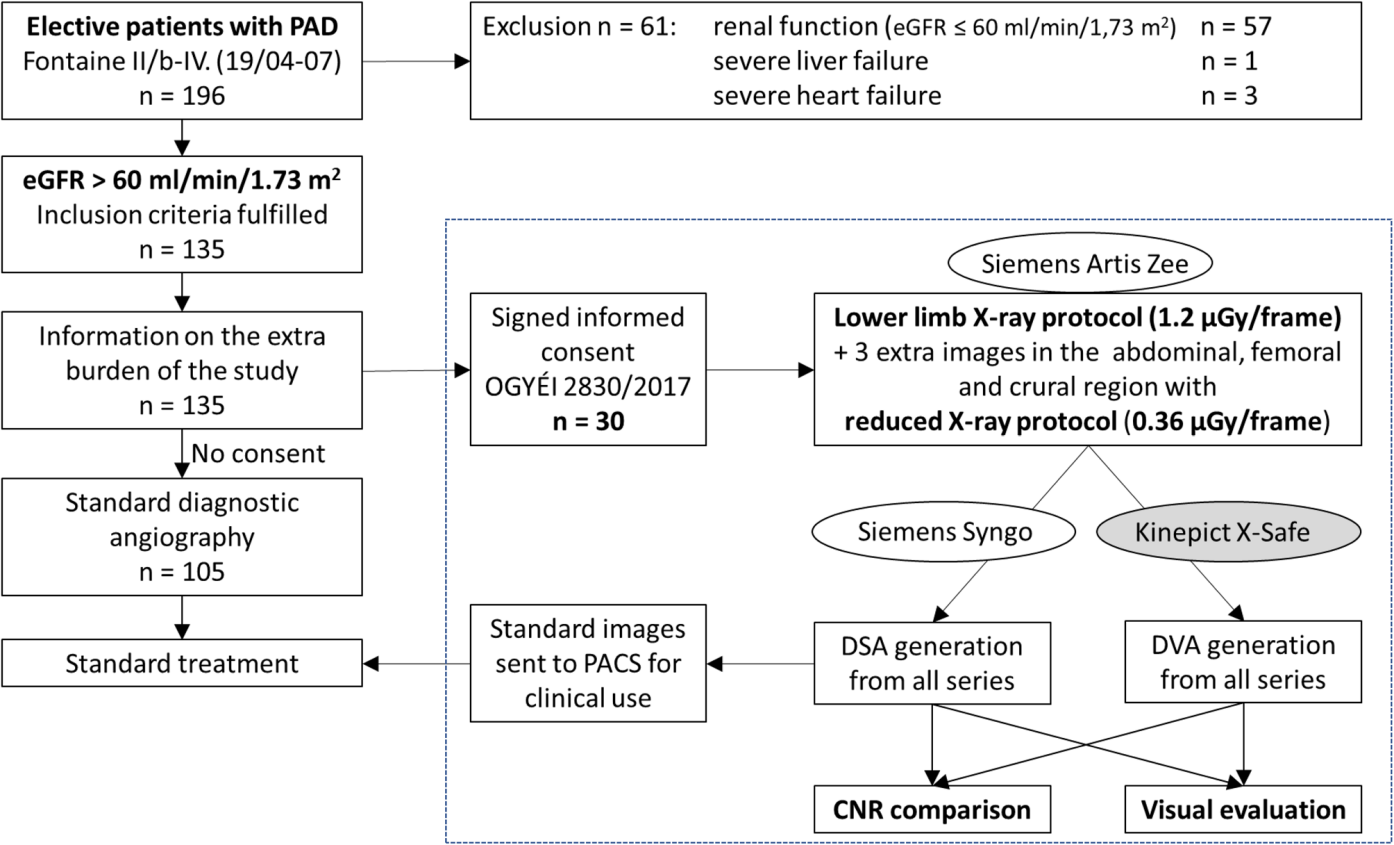
Flowchart of the study. Following the enrollment procedure, the selected patients were redirected to the study module (dashed square), where the endpoints (CNR comparison and visual evaluation) were reached in a fully anonymized manner. Although both normal and low-dose images were acquired during the examinations, only the normal dose DSA images were used for diagnostic purposes. DVA images were generated retrospectively from the raw unsubtracted series by using the Kinepict Medical Imaging Tool v.3.0 (Kinepict Health Ltd, Budapest). Both the CNR comparison and visual evaluation were performed retrospectively. PAD: Peripheral Artery Disease; DSA: Digital Subtraction Angiography; DVA: Digital Variance Angiography, PACS: Picture Archiving and Communication System; CNR: Contrast-to-Noise Ratio. OGYÉI: National Institute of Pharmacy and Nutrition.

In all enrolled patients, both normal and low-dose (70% dose/frame reduction) protocols were used for the acquisition of x-ray angiography images in three anatomical regions (Fig. 1). DSA and DVA images were generated using the Siemens Syngo (Siemens Healthcare) and the Kinepict workstation (Kinepict Health) respectively. The CNR calculation and the visual evaluation of images were performed in a retrospective manner.

### Image acquisition

Lower limb angiography was performed according to the institutional protocol using a Siemens Artis Zee with Pure system and a Syngo XWP VD11B SP2 workstation (Siemens Healthcare). A radiation saving DSA protocol, the Siemens Extremities Care was used for normal dose (1.2 µGy/frame) and its modified version for low-dose (0.36 µGy/frame) imaging. The amount of reduction (70%) was based on the previously observed CNR values^17^ and theoretical calculations.

A diagnostic catheter (Impulse, PIG 5F 125 cm; Boston Scientific) was introduced from radial access into the aorta, and after an aortography at the level of the first lumbar vertebra (abdominal image at 2 FPS) it was repositioned above the aortic bifurcation. All further injections (femoral and crural images at 1 FPS) occurred from this position. A Medrad Avanta automatized injector (Bayer) was used for injecting 7–15 ml/injection ICM (Ultravist 370, Bayer) at 9 ml/s flowrate. In all anatomical regions, a normal and a low-dose unsubtracted series was recorded.

DSA images were post-processed (brightness/contrast adjustments, pixel shift) on the Syngo workstation during the examination, and these images were used for diagnosis. DVA images were generated and post-processed retrospectively from the unsubtracted raw series using the Kinepict Medical Imaging Tool v.3.0 (Kinepict Health). The post-processed DSA and DVA images were saved for CNR calculations and visual evaluation.

### CNR analysis

For CNR measurements, regions of interest (ROI) were defined on vessels and background regions by using Image J (v.2.0.0-rc-68/1.52e, Creative Common License, NIH)^21^. The vascular and adjacent background ROI were placed in pairs (Fig. S1). The same ROI sets were used on all corresponding normal and low-dose DSA and DVA image quadruplets. ROI positions were adjusted when patient positioning or pixel shifting caused slight geometric differences. CNR values were calculated for all ROI pairs individually.

According to the following formula, wherein $$ Mean_{v}$$ and $$Mean_{b}$$ referred to mean pixel intensity values of the vascular and background ROI respectively and $$Std_{b}$$ being the background standard deviation^22^

$$ CNR = \frac{{\left| {Mean_{v} - Mean_{b} } \right|}}{{Std_{b} }} $$

CNR_DVA_/CNR_DSA_ ratios (R) for each corresponding DVA and DSA ROIs were calculated (Table 2).

**Table 2:** CNR analysis.

Region (n of ROI)	CNR values				R values		
	DVA100	DSA100	DVA30	DSA30	DVA100/DSA100	DVA30/DSA30	DVA30/DSA100
Abdominal n = 5160	22.4 (14.0–34.9)	8.2 (5.6–11.2)	15.9 (10.4–24.1)	6.3 (4.2–8.2)	2.8 (2.1–3.6)	2.8 (2.1–3.6)	2.0 (1.5–2.9)
	Wilcoxon *p* < 0.001		Wilcoxon *p* < 0.001				
		Mann–Whitney *p* < 0.001					
Femoral n = 7504	23.8 (14.0–42.2)	11.8 (7.1–17.7)	21.1 (13.0–33.5)	8.4 (5.5–12.7)	2.2 (1.6–2.8)	2.7 (1.9–3.5)	1.9 (1.4–2.5)
	Wilcoxon *p* < 0.001		Wilcoxon *p* < 0.001				
		Mann–Whitney *p* < 0.001					
Crural n = 6534	16.8 (11.2–26.0)	5.7 (3.9–8.1)	12.8 (8.2–19.8)	4.1 (3.0–6.2)	3.0 (2.3–3.8)	3.1 (2.3–4.0)	2.3 (1.7–3.0)
	Wilcoxon *p* < 0.001		Wilcoxon *p* < 0.001				
		Mann–Whitney *p* < 0.001					
Overall n = 19,198	20.9 (12.6–34.0)	8.0 (5.1–12.8)	16.0 (10.2–25.9)	6.1 (3.9–9.0)	2.6 (2.0–3.4)	2.8 (2.1–3.7)	2.0 (1.5–2.8)
	Wilcoxon *p* < 0.001		Wilcoxon *p* < 0.001				
		Mann–Whitney *p* < 0.001					

Values expressed as the median and the interquartile range (Q1–Q3). Wilcoxon signed rank test and Mann Whitney U test were used to compare paired and unpaired data sets, respectively. ROI: Region of Interest; DVA: Digital Variance Angiography, DSA: Digital Subtraction Angiography. The index defines the applied protocol, 100: normal dose, 30: low-dose.

### Visual evaluation

A blinded evaluation of images was done by three vascular surgeons (initials followed by the years of experience: P.S. 22, Z.O. 19, V.J. 6) and four interventional radiologists (B.N. 29, C.N. 8, A.P. 10, M.G. 5) working at the Heart and Vascular Center (Semmelweis University, Budapest, Hungary). Normal and low-dose DVA and DSA images were evaluated by the seven readers using the following 5-grade rating scale:

1. Poor image quality, unsuitable for diagnosis.
2. Low image quality, main vessels are distinguishable but not examinable.
3. Medium image quality, sufficient for diagnosis in the main arteries, but smaller vessels and collateralization are not examinable.
4. Good image quality, both smaller and the main vessels are examinable, suitable for everyday use.
5. Outstanding image quality, much richer in details compared to the everyday routine, makes decision-making easier.

The rating scale was implemented in a blinded and randomized web-based survey and the data were collected automatically in a data base for later processing.

### Statistical analysis

Calculations of CNR and R medians and interquartile ranges were performed using Excel 2016 (Microsoft, Redmond, WA). CNR values were compared by the Wilcoxon signed rank test or the Mann–Whitney U test (Prism 8.4.2., GraphPad), where appropriate.

For visual evaluation scores, the mean and standard error of mean (SEM), and because of the non-Gaussian distribution of data, the median and interquartile range (Q1–Q3) were also calculated. The visual scores of the corresponding DSA and DVA images generated from the same unsubtracted image series were compared by the Wilcoxon signed rank test, whereas the difference of the low-dose DVA30 and normal dose DSA100 scores was analyzed by the one-sample Wilcoxon test, to investigate the relation of these images (non-inferiority, superiority). Correlation of DVA30 and DSA100 scores was characterized by the Spearman correlation coefficient. Kendall’s W (Stata 15.0 software, StataCorp) was calculated to describe interrater agreement. Grubbs test (QuickCalcs, Graphpad) was used to detect possible outlier readers. The level of significance was set at *p* < 0.05 in all tests.

## Results

### Comparison of dose area product (DAP) values

The DSA-related DAP values were extracted from dose reports. The cumulated average (mean ± SEM) DAP of the three DSA runs (abdominal, femoral and crural) was 577 ± 38 and 186 ± 20 µGy*cm^2^ during the normal and the low-dose protocol, respectively. Thus, 70% reduction of the dose/frame parameter resulted in 68% decrease in the DSA-related DAP.

### CNR analysis

**Figure 2:**
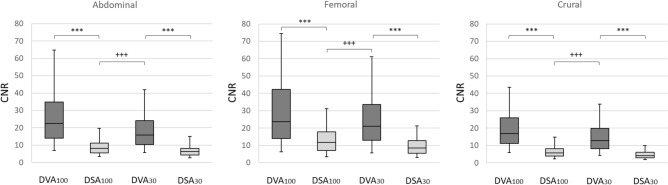
Comparison of the CNR values in three anatomical regions. The box and whisker plots show the median (line), interquartile range (box) and internal fences (whiskers) of CNR values in each group. The paired and unpaired data sets were analysed by the Wilcoxon signed rank test (***p < 0.001) and the Mann Whitney U test (+++*p* < 0.001), respectively. DSA: Digital Subtraction Angiography; DVA: Digital Variance Angiography. The index shows the applied protocol, 100: normal dose (1.2 µGy/frame), 30: low-dose (0.36 µGy/frame).

A total of 19,198 ROIs (7504 femoral, 6534 crural, 5160 abdominal) were measured on 472 images. Table 2 summarizes the results of the CNR measurements. DVA images had significantly higher CNR values (*p* < 0.001 for all comparisons, using Wilcoxon signed rank test or Mann Whitney U test, where appropriate), independently from the region and dose. The overall R (CNR_DVA_/CNR_DSA_), expressed as the median and interquartile range, was 2.6 (2.0–3.4) and 2.8 (2.1–3.7) for the normal and low-dose images, respectively. Concerning the regional data, the median R values calculated for the normal dose (CNR_DVA100_/CNR_DSA100_) and low-dose (CNR_DVA30_/CNR_DSA30_) images ranged from 2.2 to 3.0 and 2.7 to 3.1, respectively. Even the low-dose DVA30 images had better CNR than the normal dose DSA100 images, the CNR_DVA30_/CNR_DSA100_ ratio was around 2.0 (range 1.9–2.3). A consistent pattern can be observed in the data: for both image types and acquisition protocols, the femoral region had the highest and the crural region had the lowest CNR values (Fig. 2). Conversely, the femoral region had the lowest, while the crural region had the highest median R values.

### Visual evaluation

**Figure 3:**
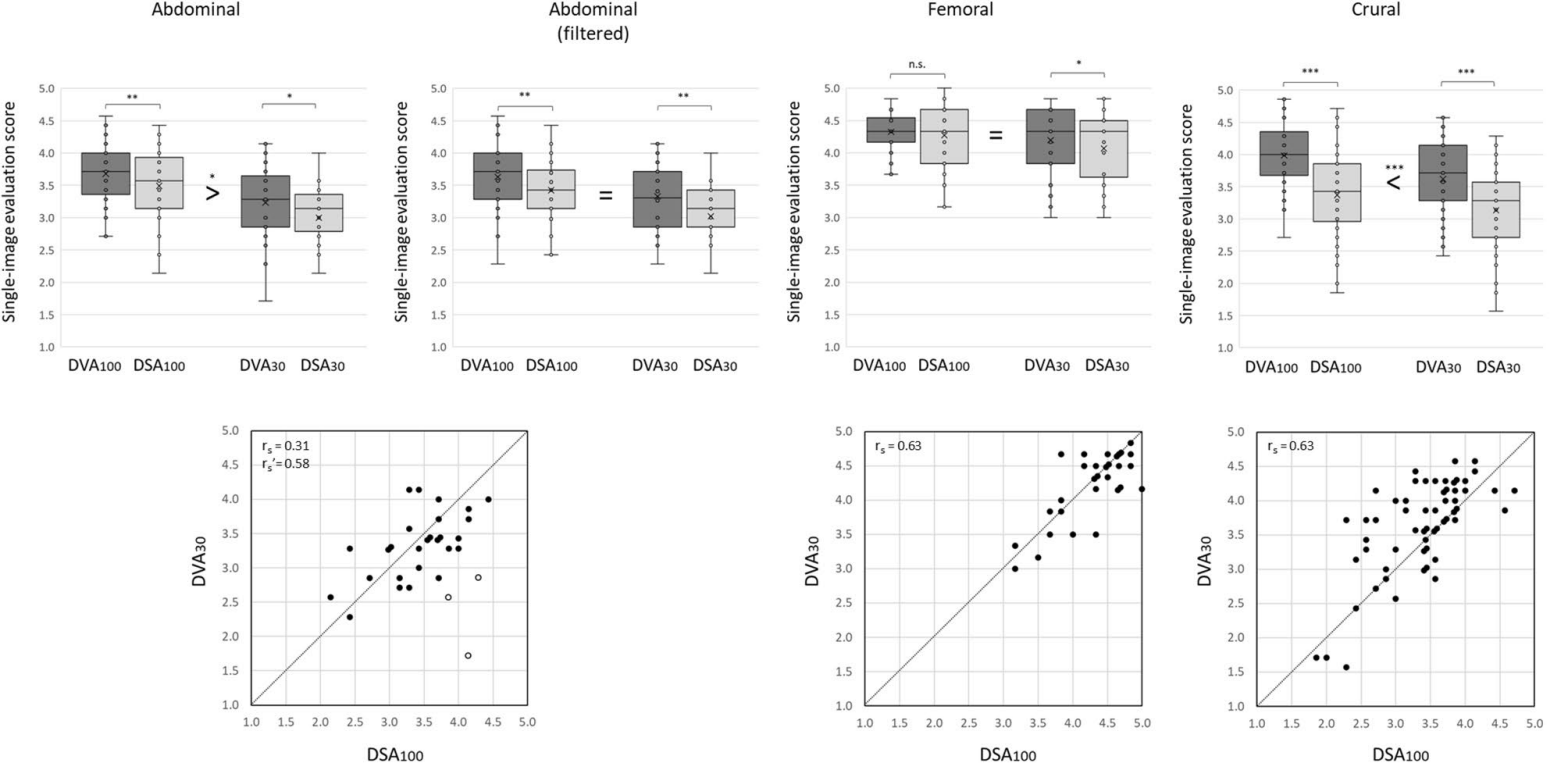
Comparison of single-image evaluation scores in three anatomical regions. The box and whisker plots (upper row) show the median (line), mean (x), interquartile range (box) and internal fences (whiskers). The image processing technologies (DVA vs DSA) were compared by the Wilcoxon signed ranked test. whereas the non-inferiority of low-dose DVA images compared to normal dose DSA images (denoted by relation signs) was analysed by the one-sample Wilcoxon test (*p < 0.05, **p < 0.01, ***p < 0.001, = identical image quality, < superior image quality). The scatter plots (lower row) show the correlation of DSA100 and DVA30 scores with the Spearman correlation coefficients (r_s_). Each point represents the mean score of a normal dose DSA100 and the corresponding low-dose DVA30 image taken from the same patient and same position. Juxtaposed points show images with identical DSA and DVA scores. Open circles in the abdominal plot represent images obtained in the presence of large amount of intestinal gases (3 patients out of 30), r_s_’ is the correlation coefficient without these points. The diagonal line separates points with higher DSA100 (below the line) or higher DVA30 scores (above the line). DSA: Digital Subtraction Angiography; DVA: Digital Variance Angiography, n.s. not significant. The index shows the applied protocol, 100: normal dose (1.2 µGy/frame), 30: low-dose (0.36 µGy/frame).

During single-image evaluation 472 normal or low-dose (DVA100, DSA100, DVA30 and DSA30, 118 in each group) images from three anatomical regions were evaluated by seven readers. Each group contained 30 abdominal, 30 femoral and 58 crural images, i.e. exactly 1 abdominal, 1 femoral and 2 crural images (right- and left-sided pixel shift) from every patient, except in two cases, where pixel shift was not necessary, therefore only one crural image was analysed. An outlier reader was identified in the femoral group by the Grubbs test (Supplementary Table S1), therefore the femoral data contain only the scores of six readers. Because of the non-Gaussian distribution of data, only non-parametric tests were used in the statistical analysis. The quality score of DSA and DVA images generated from the same unsubtracted acquisition series was compared by the Wilcoxon signed rank test. Table 3 shows the detailed results. DVA images—independently from the applied protocol or the anatomical region—always received significantly higher score than DSA images, except in the normal dose femoral group, where there was no significant difference (Fig. 3, upper panels) between the two types. The Kendall W analysis showed a moderate but significant (*p* < 0.001) interrater agreement in all regions and all protocol groups. The W value ranged from 0.51 to 0.64, except in the femoral DVA100 group, where it was 0.32 (Supplementary Table S2).

**Table 3:** Visual evaluation results.

Image	n	Mean ± SEM	Median (Q1–Q3)	Wilcoxon signed rank p	Mean/Median of (DVA30-DSA100) Wilcoxon one sample *p*
**Abdominal (n = 120)**					
DVA100	30	3.67 ± 0.10	3.71 (3.43–4.00)	**0.003**	− 0.26 ± 0.12/ − 0.29 (− 0.54–0.25) **0.036**
DSA100	30	3.49 ± 0.10	3.57 (3.18–3.86)		
DVA30	30	3.23 ± 0.10	3.29 (2.86–3.54)	**0.013**	
DSA30	30	3.00 ± 0.10	3.07 (2.86–3.29)		
**Abdominal, after exclusion of patients with excessive intestinal gases (n = 108)**					
DVA100	27	3.63 ± 0.10	3.71 (3.36–4.00)	**0.002**	− 0.10 ± 0.09/ − 0.14 (− 0.43–0.29) 0.350
DSA100	27	3.42 ± 0.10	3.43 (3.14–3.72)		
DVA30	27	3.33 ± 0.09	3.31 (2.93–3.64)	**0.001**	
DSA30	27	3.02 ± 0.10	3.14 (2.86–3.36)		
**Femoral (n = 120)**					
DVA100	30	4.32 ± 0.06	4.33 (4.21–4.50)	0.390	− 0.08 ± 0.06/0.00 (− 0.17–0.13) 0.435
DSA100	30	4.27 ± 0.09	4.33 (3.88–4.67)		
DVA30	30	4.19 ± 0.09	4.33 (3.88–4.63)	**0.020**	
DSA30	30	4.07 ± 0.11	4.33 (3.75–4.46)		
**Crural (n = 232)**					
DVA100	58	3.99 ± 0.07	4.00 (3.71–4.29)	** < 0.001**	0.25 ± 0.07/0.21 (0.00–0.68) < **0.001**
DSA100	58	3.37 ± 0.08	3.43 (3.00–3.82)		
DVA30	58	3.62 ± 0.09	3.71 (3.29–4.14)	** < 0.001**	
DSA30	58	3.14 ± 0.09	3.29 (2.71–3.57)		

Readers evaluated in total 472 images by using a 5-grade Likert scale: ranging from poor (1) to outstanding (5) image quality (for details see The Materials and Methods section). Values are expressed as the mean ± SEM, and the median and the interquartile range (Q1–Q3). Data were analysed by the Wilcoxon signed rank test (DVA vs DSA) or the Wilcoxon one sample test (non-inferiority analysis between DVA30 and DSA100). Bold: significant difference, *p* < 0.05. DVA: Digital Variance Angiography, DSA: Digital Subtraction Angiography. The index defines the applied protocol, 100: normal dose, 30: low-dose. SEM: Standard Error of Mean.

The key question of this study was, whether low-dose DVA is able to produce non-inferior image quality compared to normal dose DSA, therefore the further analysis concentrated on these two groups. This question was investigated by a correlation analysis and a non-inferiority analysis between the normal dose DSA100 and low-dose DVA30 scores. The Spearman correlation analysis (Fig. 3, lower row) showed a strong positive correlation in the femoral and crural regions (r_s_ = 0.63, *p* < 0.001 in both cases), but there was only a weak and non-significant positive correlation (r_s_ = 0.31, p = 0.101) in the abdominal region. The abdominal scatter plot showed three strikingly outlying points (marked with open circles in Fig. 3) with huge difference in the DSA100 and DVA30 scores, having much higher DSA100 scores. Meticulous examination of the corresponding images revealed the presence of large amount of intestinal gases (see Supplementary Fig. S2) in these cases. The correlation without these points dramatically increased to a highly significant, moderately positive level also in the abdominal region (r_s_ = 0.58, *p* = 0.001).

For addressing the non-inferiority question, the difference of DVA30 and DSA100 scores was statistically analysed by the ‘one-sample’ Wilcoxon test to see, whether these values are significantly different from zero, the ‘equal quality’ point. There was no statistical difference in the femoral region (mean ± SEM of DVA30-DSA100 = − 0.08 ± 0.06, range from − 0.83 to 0.83, *p* = 0.435), but the results showed significant difference in the abdominal (mean ± SEM of DVA30-DSA100 = − 0.26 ± 0.12, range from − 2.43 to 0.86, DSA100 is better than DVA30, *p* = 0.036) and the crural region (0.25 ± 0.07 (range from − 0.71 to 1.43, DVA30 is better than DSA100, *p* < 0.001). However, after exclusion of patients with intestinal gases, the difference in the abdominal region decreased to − 0.10 ± 0.09 (range from − 0.86 to 0.86, *p* = 0.350) and was no longer significant. (Table 3).

**Figure 4:**
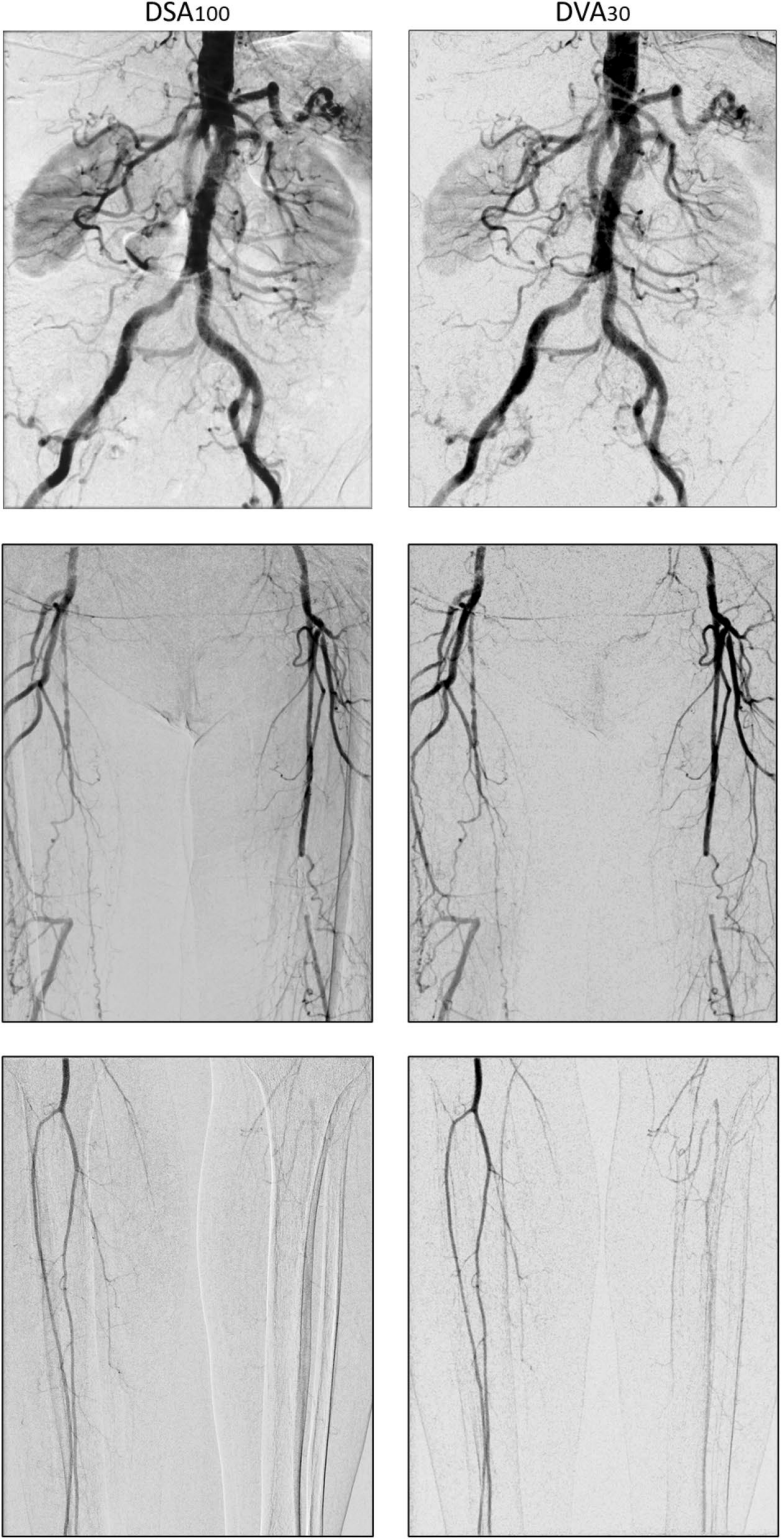
Comparison of representative normal dose (1.2 µGy/frame) DSA100 (left column) and low-dose (0.36 µGy/frame) DVA30 (right column) images obtained in the abdominal (upper row), femoral (middle row) and crural (lower row) region. The corresponding Image pairs were taken from the same patient and same direction in two consecutive runs, but the different regions belong to different patients. Brightness/contrast adjustments and pixel shift were applied to DSA and DVA images using the Siemens Syngo and the Kinepict workstation, respectively. Abbreviations: DSA: Digital Subtraction Angiography; DVA: Digital Variance Angiography.

Figure 4 illustrates the capabilities of DVA by showing representative DSA100 and DVA30 image pairs from the three anatomical regions from three different patients.

## Discussion

Our paper is the first report on the radiation dose management capability of DVA. The major aim of this study was to clarify whether the quality reserve of DVA can be used to reduce radiation exposure in lower limb X-ray angiography, i.e. whether the low-dose DVA (70% dose/frame reduction) can provide non-inferior image quality compared to the normal dose DSA. In agreement with previous findings^17,18^, DVA produced consistently higher (two to threefold) CNR than DSA. The highest ratios (3.0–3.1) were observed in the crural region, where the CNR values were the lowest in the three anatomical regions, indicating that the quality difference between DSA and DVA is higher in regions where the image quality is generally worse. The visual evaluation data were in line with these observations, since DVA images received significantly higher scores in all anatomical regions and protocols (except the normal dose femoral group, where there was no significant difference), and the advantage of DVA was the most emphasized in the crural region, where even the low-dose DVA30 images could outperform the normal dose DSA100 images. This finding reveals the special advantage of DVA in visualizing small vessel areas. In the femoral region there was no significant difference between DVA30 and DSA100, and this was the only region where even the weakest image type, DSA30 received an average score above 4 (representing the daily routine quality suitable for diagnosis), suggesting that probably a higher radiation dose reduction could have been reached here. DVA showed the weakest performance in the abdominal region, where the analysis of score differences indicated that DSA100 provided significantly better image quality than DVA30. Nevertheless, a thorough analysis revealed, that this difference arose from 3 patients with excessive intestinal gases. Without these patients, there was no statistical difference between the two image types in the abdominal region, and DVA30 provided similar image quality to DSA100. These data indicate that DVA, compared to normal dose DSA images, was able to provide non-inferior image quality in the femoral and (with some caveats) in the abdominal regions, and superior image quality in the crural region at a markedly reduced radiation dose, resulting in 68% reduction in the DSA-related DAP in lower limb X-ray angiography. Regarding the substantial (50–90%) contribution of DSA-related DAP to the total procedural radiation load^14,15^, the use of DVA might provide a considerable (30–60%) radiation exposure reduction in endovascular lower limb interventions.

Our results have major implications in radiation protection. As the DVA technology is implemented in a platform-independent software, it can be combined with any angiography system. Since its dose management capabilities are based on a unique image processing algorithm, the beneficial effects of DVA will be additive and can further enhance the efficacy of any existing hardware- or software-based radiation protection solutions, like in our study, where we achieved about 70% radiation reduction compared to an already reduced radiation protocol, Extremities Care. For these reasons DVA can be a very effective tool to further reduce the radiation burden of patients and the medical staff, thereby the safety of lower limb endovascular interventions might be greatly enhanced.

Our study has some limitations. As DVA is based on the principles of kinetic imaging^16^, the technology is especially sensitive to motion artifacts, that are not corrigible by pixelshift, therefore the bowel movements and gases might significantly deteriorate its image quality. In our study group we did not apply any pre-treatment to reduce intestinal peristaltic movements or gases, but even under these conditions, disturbing interference was experienced only in 10% of the patients. Obviously, this proportion could have been reduced by an appropriate pre-operative preparation, but this is not part of the standard care in lower limb interventions, because the pathology and consequently the site of intervention is located mainly in the femoropopliteal or crural regions. Nevertheless, the use of DVA in other clinical settings focusing on the abdominal region (e.g. embolization procedures) might require a careful preparation to minimize moving artifacts.

Another limitation might be the relatively low number of patients and the retrospective image processing. However, it should be emphasized that, because of ethical considerations, this study was intentionally planned as a small cohort proof-of-concept investigation, to verify the feasibility of radiation exposure reduction. Based on the positive results, we have already initiated a prospective study on a larger cohort involving more than a hundred patients, where the whole lower limb procedure is performed at a reduced radiation dose, and the DVA technology is used in real-time setting in the operating room, as described recently^23^. A further consideration is that the study verified the dose management capability of DVA only in lower limb interventions, and the results cannot be transferred automatically to other areas or indications without appropriate validation studies. The extent of radiation dose reduction will vary depending on the technical conditions (angiography system, applied dose management solutions) and the anatomical properties of the target region.

In conclusion, our data show that the use of DVA instead of DSA allows a very substantial reduction of radiation exposure in lower limb X-ray angiography, thereby this technology can increase the safety of the lower limb endovascular procedures by reducing the risk of radiation-induced side effects and long-term damages. The results warrant further clinical studies to investigate the dose management capabilities of DVA in other clinical settings (like embolization or neuroradiography), and to validate the benefits in a larger population.

## Supplementary Information

Supplementary Information.

## Data Availability

Study protocols, ethical approval documents, fully anonymised images, CNR calculations and visual evaluation raw data are available on request at the Heart and Vascular Center and at the head office of Kinepict Health Ltd.
