# Supplementary material for: Digital variance angiography allows about 70% decrease of DSA-related radiation exposure in lower limb X-ray angiography
Source: Sci Rep. 2021 Nov 8;11:21790. doi: 10.1038/s41598-021-01208-3 (PMC8575921; doi:10.1038/s41598-021-01208-3)
Supplement: Supplementary file 1 — Supplementary Information. [file 41598_2021_1208_MOESM1_ESM.pdf]

DIGITAL VARIANCE ANGIOGRAPHY ALLOWS ABOUT 70 % DECREASE OF DSA-RELATED RADIATION EXPOSURE IN LOWER LIMB X-RAY ANGIOGRAPHY

Marcell Gyánó[1,2,5], Márton Berczeli[1,2,5], Csaba Csobay-Novák[1], Dávid Szöllősi[2,3], Viktor I. Óriás[1,2], István Góg[2,4], János P. Kiss[2], Dániel S. Veres[3], Krisztián Szigeti[2,3], Szabolcs Osváth[2,3], Ákos Pataki[1], Viktória Juhász[1], Zoltán Oláh[1], Péter Sótonyi[1,6], Balázs Nemes[1,6]

[1] The Heart and Vascular Center, Semmelweis University, Budapest, Hungary

[2] Kinepict Health Ltd, Budapest, Hungary

[3] Department of Biophysics and Radiation Biology, Semmelweis University, Budapest, Hungary

[4] Department of Vascular Surgery, Hungarian Defence Forces Medical Centre, Budapest, Hungary

Corresponding author: Prof Péter Sótonyi, MD, PhD, sotonyi.peter1@med.semmelweis-univ.hu,, tel: +36 1 458 6734, The Heart and Vascular Center, Semmelweis University, Városmajor utca 68, 1122 Budapest, Hungary

Joint authorships:

[5]Marcell Gyánó and Márton Berczeli contributed equally to the paper as first authors

[6]Péter Sótonyi and Balázs Nemes contributed equally to the paper as last authors.

SUPPLEMENTARY INFORMATION

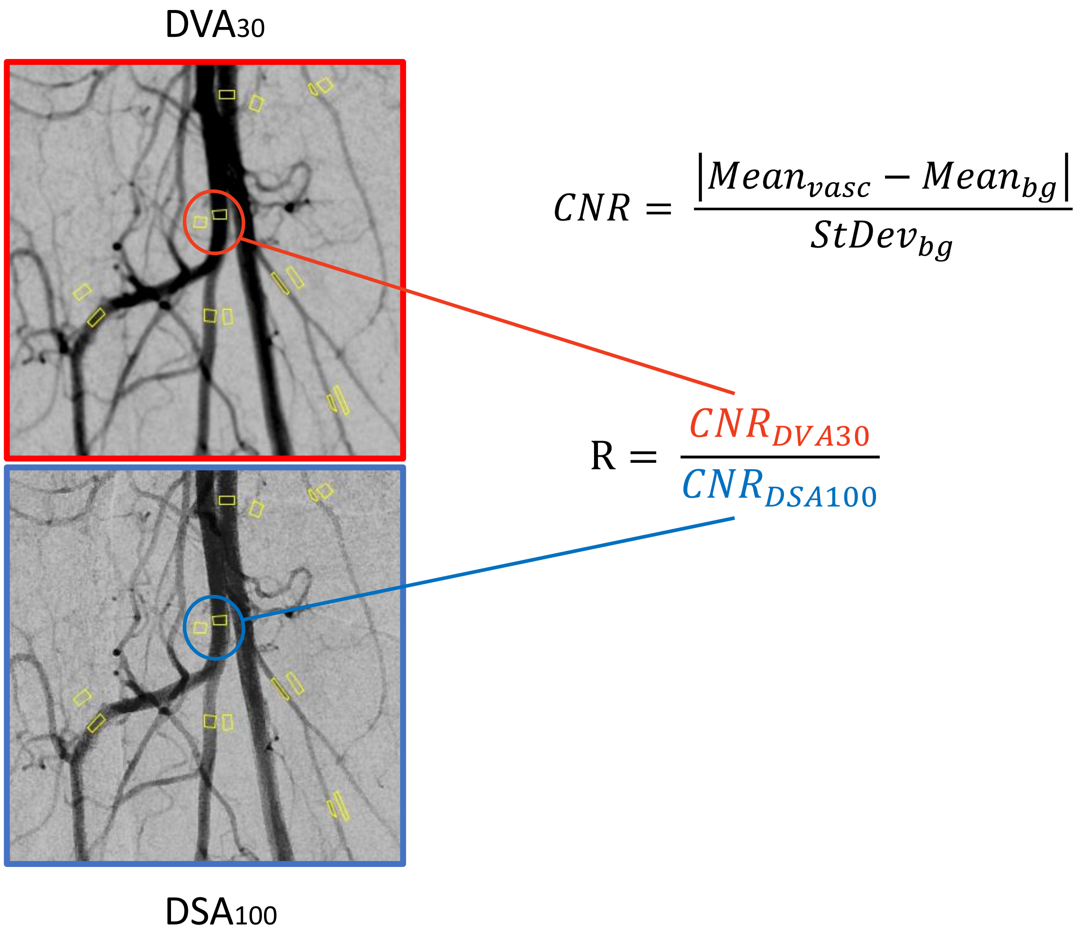

$$CNR = \frac{|Mean_{vasc} - Mean_{bg}|}{StDev_{bg}}$$

$$R = \frac{CNR_{DVA30}}{CNR_{DSA100}}$$

Figure S1. Calculation of the contrast-to-noise ratio and the R value. Yellow rectangles show the manually selected region-of interest (ROI) pairs, a vascular and a background area. The same ROI pairs were applied to each corresponding image. The R value is the ratio of the CNR of different images. For further details, see the Materials and Methods section. DVA: Digital Variance Angiography; DSA: Digital Subtraction Angiography.

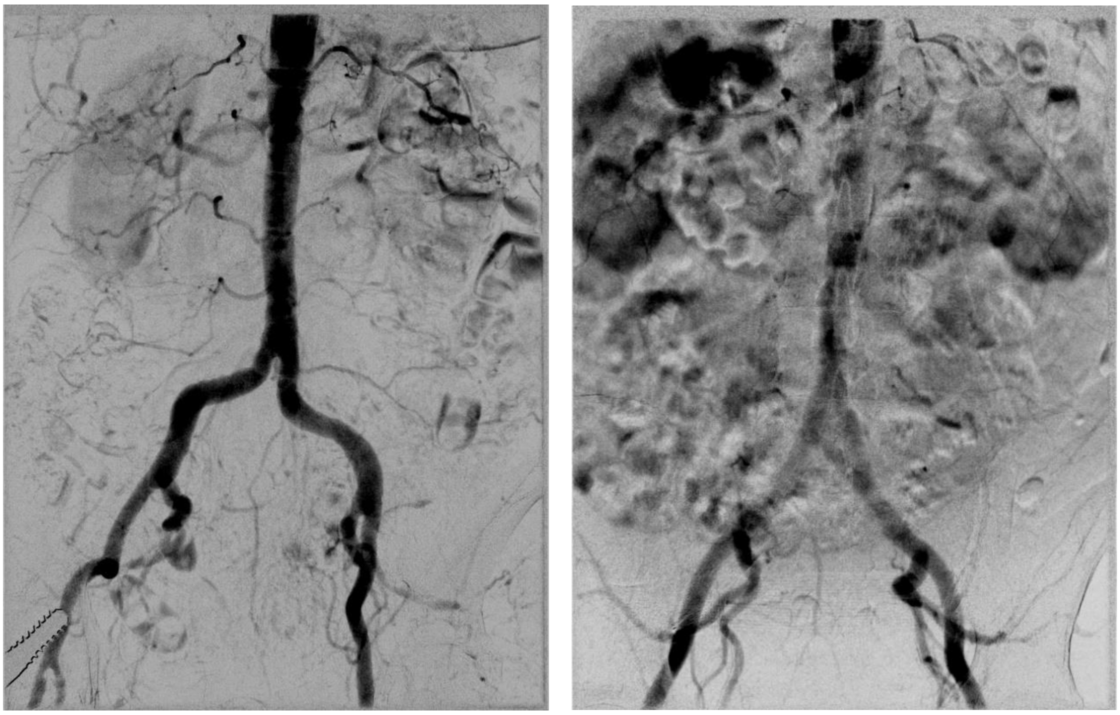

Figure S2. Comparison of abdominal low-dose DVA_{30} images in normal patients (left panel) and patients with large amount of intestinal gases (right panel). The images illustrate the difference between regular and outlier abdominal images (see Figure 3). In the study, outlier images represented only 10 % (3/30) of the total abdominal images. DVA: Digital Variance Angiography.

crural	DSA100	DVA30	DVA30-DSA100
VJ	3.86	3.88	**0.02**
PS	3.45	3.81	**0.36**
BN	3.55	3.71	**0.16**
MG	3.17	3.71	**0.53**
ZO	3.45	3.31	**-0.14**
CC	3.36	3.86	**0.50**
AP	2.74	3.07	**0.33**

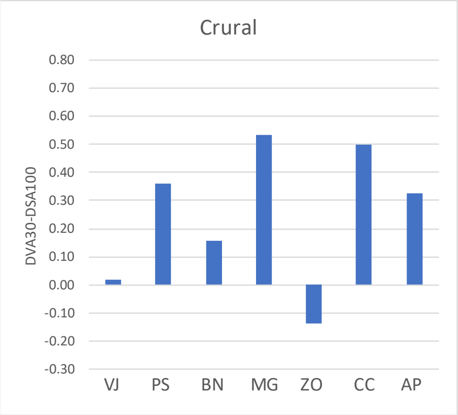

Mean: 0.2514
SD: 0.2492
# of values: 7
Outlier detected? No
Significance level: 0.05 (two-sided)
Critical value of Z: 2.01997

Value	Z	Significant Outlier?
0.02	0.9288	
0.36	0.4357	
0.16	0.3669	
0.53	1.118	
-0.14	1.571	Furthest from the rest, but not significant
0.5	0.9976	
0.33	0.3153	

femoral	DSA100	DVA30	DVA30-DSA100
VJ	4.37	4.23	**-0.13**
PS	4.43	4.30	**-0.13**
BN	4.23	4.17	**-0.07**
MG	4.37	4.43	**0.07**
ZO	4.30	3.53	**-0.77**
CC	4.30	4.20	**-0.10**
AP	3.93	3.83	**-0.10**

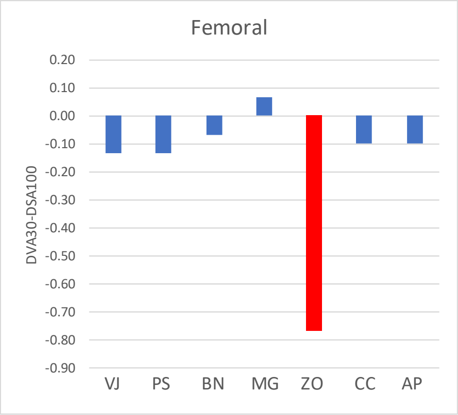

Mean: -0.1757
SD: 0.2709
# of values: 7
Outlier detected? Yes
Significance level: 0.05 (two-sided)
Critical value of Z: 2.01997

Value	Z	Significant Outlier?
-0.13	0.1687	
-0.13	0.1687	
-0.07	0.3902	
0.07	0.907	
-0.77	2.1936	Significant outlier. P < 0.05
-0.1	0.2795	
-0.1	0.2795	

abdomin.	DSA100	DVA30	DVA30-DSA100
VJ	3.77	3.47	**-0.30**
PS	3.67	3.33	**-0.33**
BN	3.67	3.27	**-0.40**
MG	3.50	3.47	**-0.03**
ZO	2.90	2.40	**-0.50**
CC	3.43	3.37	**-0.07**
AP	3.50	3.33	**-0.17**

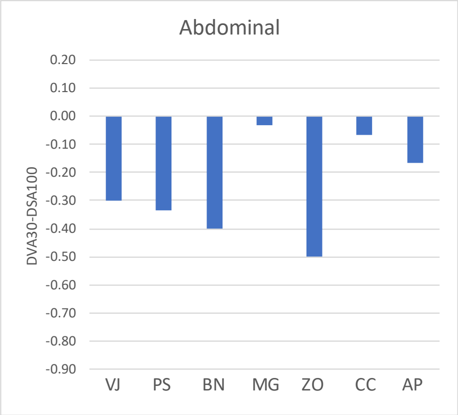

Mean: -0.2571
SD: 0.1736
# of values: 7
Outlier detected? No
Significance level: 0.05 (two-sided)
Critical value of Z: 2.01997

Value	Z	Significant Outlier?
-0.3	0.2469	
-0.33	0.4198	
-0.4	0.8231	
-0.03	1.3087	
-0.5	1.3993	Furthest from the rest, but not significant
-0.07	1.0782	
-0.17	0.5021	

abd. filt.	DSA100	DVA30	DVA30-DSA100
VJ	3.70	3.56	**-0.14**
PS	3.56	3.41	**-0.15**
BN	3.67	3.33	**-0.34**
MG	3.33	3.59	**0.26**
ZO	2.81	2.52	**-0.29**
CC	3.33	3.44	**0.11**
AP	3.48	3.44	**-0.04**

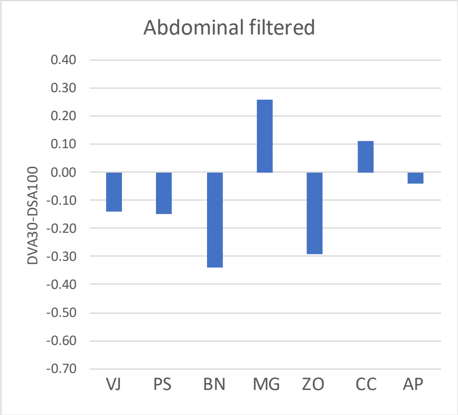

Mean: -0.0843
SD: 0.2135
# of values: 7
Outlier detected? No
Significance level: 0.05 (two-sided)
Critical value of Z: 2.01997

Value	Z	Significant Outlier?
-0.14	0.261	
-0.15	0.3079	
-0.34	1.198	
0.26	1.6129	Furthest from the rest, but not significant
-0.29	0.9637	
0.11	0.9102	
-0.04	0.2075	

Table S1. Outlier analysis. The difference between low-dose DVA_{30} and normal dose DSA_{100} scores was calculated for each reader individually in the three anatomical regions. Filtered abdominal data show the results without the three patients with excessive intestinal gases. The Grubbs test revealed an outlier reader in the femoral region (red bar), therefore his femoral scores were not included in the final analysis. DSA: Digital Subtraction Angiography; DVA: Digital Variance Angiography. The index shows the applied protocol, 100: normal dose (1.2 µGy/frame), 30: low-dose (0.36 µGy/frame).

	abdominal	femoral	crural
DVA_{100}	0.5476	0.3199	0.5576
DSA_{100}	0.5723	0.5666	0.6424
DVA_{30}	0.5185	0.5112	0.5763
DSA_{30}	0.5513	0.5635	0.6345

Table S2. Interrater agreement analysis. The Kendall W was used to describe the concordance between readers. All W values were highly significant ($p < 0.001$). DSA: Digital Subtraction Angiography; DVA: Digital Variance Angiography, n.s. not significant. The index shows the applied protocol, 100: normal dose (1.2 µGy/frame), 30: low-dose (0.36 µGy/frame).
